# Predictive Value of Interferon-Lambda Gene Polymorphisms for Treatment Response in Chronic Hepatitis C

**DOI:** 10.1371/journal.pone.0112592

**Published:** 2014-11-13

**Authors:** Simone Susser, Eva Herrmann, Christian Lange, Nabila Hamdi, Tobias Müller, Thomas Berg, Dany Perner, Stefan Zeuzem, Christoph Sarrazin

**Affiliations:** 1 Medical Department 1, Goethe-University Hospital Frankfurt/Main, Frankfurt, Germany; 2 Institute of Biostatistics and Mathematical Modeling, Goethe-University Hospital Frankfurt/Main, Frankfurt, Germany; 3 Department of Pharmacology and Toxicology, German University in Cairo, Cairo, Egypt; 4 Clinic for Gastroenterology, Section Hepatology, University Hospital Leipzig, Leipzig, Germany; University of Pisa, Italy

## Abstract

**Background:**

*IL28B* gene polymorphism is the best baseline predictor of response to interferon alfa-based antiviral therapies in chronic hepatitis C. Recently, a new *IFN-L4* polymorphism was identified as first potential functional variant for induction of *IL28B* expression. Individualization of interferon alfa-based therapies based on a combination of *IL28B/IFN-L4* polymorphisms may help to optimize virologic outcome and economic resources.

**Methods:**

Optimization of treatment outcome prediction was assessed by combination of different *IL28B* and *IFN-L4* polymorphisms in patients with chronic HCV genotype 1 (n = 385), 2/3 (n = 267), and 4 (n = 220) infection treated with pegylated interferon alfa (PEG-IFN) and ribavirin with (n = 79) or without telaprevir. Healthy people from Germany (n = 283) and Egypt (n = 96) served as controls.

**Results:**

Frequencies of beneficial *IL28B* rs12979860 C/C genotypes were lower in HCV genotype 1/4 infected patients in comparison to controls (20–35% vs. 46–47%) this was also true for ss469415590 TT/TT (20–35% vs. 45–47%). Single interferon-lambda SNPs (rs12979860, rs8099917, ss469415590) correlated with sustained virologic response (SVR) in genotype 1, 3, and 4 infected patients while no association was observed for genotype 2. Interestingly, in genotype 3 infected patients, best SVR prediction was based on *IFN-L4* genotype. Prediction of SVR with high accuracy (71–96%) was possible in genotype 1, 2, 3 and 4 infected patients who received PEG-IFN/ribavirin combination therapy by selection of beneficial *IL28B* rs12979860 C/C and/or ss469415590 TT/TT genotypes (p<0.001). For triple therapy with first generation protease inhibitors (PIs) (boceprevir, telaprevir) prediction of high SVR (90%) rates was based on the presence of at least one beneficial genotype of the 3 IFN-lambda SNPs.

**Conclusion:**

*IFN-L4* seems to be the best single predictor of SVR in genotype 3 infected patients. For optimized prediction of SVR by treatment with dual combination or first generation PI triple therapies, grouping of interferon-lambda haplotypes may be helpful with positive predictive values of 71–96%.

## Introduction

With estimated 150 million people chronically infected worldwide and 3–4 million new infections each year [1], hepatitis C virus (HCV) infection is one of the major causes for liver cirrhosis and subsequent development of hepatocellular carcinoma. This often leads to liver failure and thus to liver transplantation.

A large number of direct acting antiviral agents (DAAs) currently are investigated in clinical studies for improvement of treatment response, better tolerability, reduction of treatment duration and establishment of an interferon-free treatment option [2]. Recently, in some countries combination therapies with the nucleoside inhibitor sofosbuvir were approved as a first step to an interferon-free treatment of chronic hepatitis C [3]–[6]. However, in many countries triple therapies with HCV NS3 protease inhibitors telaprevir (TVR) or boceprevir (BOC) and/or dual combination of pegylated interferon alfa (PEG-IFN) and ribavirin will remain the standard of care for the next years before newer DAA containing regimens are approved or reimbursed [7]–[10]. Also due to high costs associated with the use of DAAs, strategies are explored to identify easy to treat patients with excellent chances of sustained virologic response (SVR) with a short treatment of PEG-IFN-based dual or triple combination therapies [11], [12].

Interleukin 28B *(IL28B* also named *IFN-L3)* gene polymorphisms are the most important predictors for SVR to PEG-IFN/ribavirin combination therapy in genotype 1/4 and to a lesser extend also in genotype 2/3 infected patients as well as for TVR and BOC-based triple-therapies in treatment-naïve patients [7]–[10], [13]–[23].

Recently, a new variant in the CpG region upstream of *IL28B* was observed, with the assumption to be the functional variant leading to expression of *IL28B* and *IP-10*. This so called TT/ΔG polymorphism (ss469415590) is described to be a better predictor of HCV clearance than *IL28B* rs12979860 CC genotype [24]. In addition, it was postulated that the ΔG-allele leads to expression of a new interferon gene – *IFN-L4* – which is associated with impaired clearance of HCV. Expression of interferon-stimulated genes (ISGs) could be induced by overexpression of *IFN-L4* in a hepatoma cell line [25].

In the present study, the clinical value of the new SNP ss469415590 TT/ΔG on treatment outcome prediction to dual- and triple-therapy in HCV genotype 1, 2, 3, and 4 infected patients in comparison to established *IL28B* SNPs was investigated with the aim to establish an algorithm for potential selection of IFN-based therapies in subgroups of patients with excellent SVR chances.

## Methods

### Patients

Patients chronically infected with HCV genotype 1 from a German multicenter study (INDIV-2) (n = 385) [26] and consecutive patients infected with HCV genotype 1 (n = 81), genotype 2/3 (n = 270; genotype 2 n = 79; genotype 3, n = 191) and genotype 4 (n = 212) who presented at tertiary hepatology referral centers at University Hospitals in Frankfurt/Main, Homburg/Saar, Berlin, Germany and Cairo, Egypt, between 1998 and 2012 were enrolled. Antiviral therapy with known virologic outcome was performed in 367/385 genotype 1 dual-therapy, 75/81 genotype 1 triple-therapy, 201/270 genotype 2/3, and 197/197 genotype 4 patients. Reasons for missing outcome information were screening failures, discontinuation of therapy (non-virologic failures, e.g. due to side-effects) or ongoing follow-up. These patients were excluded from further analysis as we tried to optimize prediction of the virologic course of therapy. A summary of patients’ characteristics is shown in Table 1.

**Table 1:** Virologic and histological characteristics of patients infected with HCV genotype 1 (dual and triple therapy), 2, 3, and 4.

Variable	GT1(d)	GT1(t)	GT2	GT3	GT4
	(n = 385)	(n = 81)	(n = 79)	(n = 191)	(n = 220)
SVR	203 (55%)	52 (69%)	53 (89%)	105 (75%)	137 (62%)
Relapse	74 (20%)	4 (5%)	5 (8%)	19 (13%)	44 (20%)
Non-response	90 (25%)	19 (25%)	2 (3%)	17 (12%)	39 (18%)
Metavir fibrosis stage					
** **F0–1	227 (65%)	23 (36%)	22 (47%)	50 (39%)	n. a.
** **F2	74 (21%)	8 (12%)	10 (21%)	41 (32%)	n. a.
** **F3–4	48 (14%)	33 (52%)	15 (32%)	37 (29%)	n. a.

GT1(d), HCV genotype 1 infected patients receiving dual-therapy (PEG-IFN + ribavirin); GT1(t), HCV genotype 1 infected patients receiving triple-therapy (TVR+PEG-IFN + ribavirin).

GT, genotype.

SVR, sustained virologic response.

n. a., not applicable.

Treatment consisted of standard IFN 2a/b 3 million IU three times per week, PEG-IFN 2a 180 µg per week or PEG-IFN 2b 1.5 µg per kg body weight (bw) and week in combination with 600 to 1400 mg ribavirin per day according to body weight for a duration of 24 to 72 weeks. Treatment duration was 24 weeks in genotype 2/3 infected patients and 48 weeks in patients with genotype 4 infection. For genotype 1 infected patients an individualized treatment duration between 24 and 72 weeks was calculated based on early viral kinetics as described previously [26]. Triple-therapy consisted of telaprevir 750 mg three times daily plus PEG-IFN 2a 180 µg per week in combination with ribavirin 1000 (<75 kg bw) or 1200 mg (≥75 kg bw) every day for 12 weeks with subsequent dual-therapy for additional 12 or 36 weeks according to virologic response at treatment weeks 4 and 12. In treatment naïve or relapse patients with liver cirrhosis and undetectable HCV RNA at weeks 4 and 12 of triple therapy treatment was shortened to 24 weeks.

SVR was defined as HCV RNA negativity by a sensitive assay (detection limit <50 IU/mL) at least 24 weeks after termination of antiviral dual-therapy or 12 weeks after termination of triple-therapy.

Virologic relapse was defined as HCV RNA undetectable at the end-of-treatment but positive thereafter and virologic non-response as HCV RNA detectability throughout the entire therapy of at least 24 weeks or less than 2log_10_ decline of HCV RNA concentration until week 12 of treatment.

In addition, random samples of healthy European (n = 283) and Egyptian (n = 96) volunteers were enrolled as controls.

Analyses on viral load were limited to patients with available HCV RNA concentration at baseline before initiation of antiviral therapy. HCV RNA viral load was measured by Cobas Amplicor Monitor 2.0, Cobas TaqMan HCV (Roche Diagnostics, Mannheim, Germany), Siemens Versant Quantitative bDNA 3.0 (Siemens Diagnostics, Eschborn, Germany) or National Genetics Institute SuperQuant (NGI, Culver City, CA, USA) assays.

HCV genotyping was performed by a reverse hybridisation assay (Versant InnoLipa, HCV assays vs1 and vs2, Innogenetics, Zwijnaarde, Belgium and Siemens Diagnostics, Eschborn, Germany) for HCV genotypes 1, 2, and 3, or through Sanger sequencing of a 288 bp region in the NS5B gene for HCV genotype 4 samples [27].

Co-infection with hepatitis B virus (HBV) and human immunodeficiency virus (HIV) was excluded in all patients and controls by standard serological tests (HBs antigen, HIV-1/2 antibodies).

Histological results of liver biopsies were classified by local pathologists at the different study sites according to internationally standardized criteria. For better comparison between the different local pathologists the individual fibrosis stage was documented as stage 0–1, stage 2 or stage 3–4 (i.e., absence or minimal fibrosis, moderate fibrosis or advanced fibrosis/presence of cirrhosis according to the Metavir scoring system F1–4).

All clinical studies were approved by local ethics committees, Ethik-Kommission der Ärztekammer des Saarlandes, Klinisches Ethik-Komitee Universitätsklinikum Frankfurt, and Ethikkommission der Charité - Universitätsmedizin Berlin. The experiments concerning the HCV genotype 4 infected patients were performed in compliance with the guidelines of the institutional review board of Kasr-El-Aini Medical School in Cairo University. Written informed consent was obtained from all patients and healthy controls and the study was performed in accordance with provisions of the Declaration of Helsinki and Good Clinical Practice guidelines.

### DNA collection and extraction

Blood was collected into EDTA tubes. Genomic DNA was extracted using the QIAamp DNA Blood Mini Kit (Qiagen, Hilden, Germany) according to the manufacturer’s instructions. DNA quality was assessed by calculating the absorbance ratio OD_260 nm/280 nm_ using NanoDrop model ND-1000 (PeqLab, Erlangen).

### Genotyping of IFN-lambda

#### IL28B (IFN-L3)

Genotypes of rs12979860 and rs8099917 were determined using a custom-designed TaqMan SNP Genotyping assay for rs12979860 (forward primer: rs12979860_F GCCTGTCGTGTACTGAACCA; reverse primer: rs12979860_R GCGCGGAGTGCAATTCAAC; probes: rs12979860_V VIC-TGGTTCGCGCCTTC-NFQ and rs12979860_M FAM-CTGGTTCACGCCTTC -NFQ) and an inventoried TaqMan SNP Genotyping assay for rs8099917 (forward primer: C__11710096_10_F; reverse primer: C__11710096_10_R; probes: C__11710096_10_V VIC NFQ C__11710096_10_M FAM NFQ targeting TTTTGTTTTCCTTTCTGTGAGCAAT[G/T]TCACCCAAATTGGAACCATGCTGTA on chromosome 19q13). Data for *IL28B* SNPs have been reported in part in a previous publication [16].

#### IFN-L4

The variants of ss469415590 were diagnosed using a custom-designed TaqMan SNP Genotyping assay (forward primer: ss469415590IFNL4_F GCCTGCTGCAGAAGCAGAGAT; reverse primer: ss469415590IFNL4_R GCTCCAGCGAGCGGTAGTG; probes: ss469415590IFNL4_V VIC-ATCGCAGAAGGCC-NFQ and ss469415590IFNL4_M FAM-ATCGCAGCGGCCC-NFQ).

All reactions were set up with 1 µL of isolated gDNA and TaqMan Genotyping Master Mix, the genotyping ran on a StepOnePlus instrument (Life Technologies GmbH, Darmstadt, Germany). Genotyping was performed at Goethe-University Hospital, Frankfurt, Germany.

### Statistical analysis

Predictors for SVR were assessed by multivariate logistic regression analysis. Multivariate analysis included all significant parameters from nonparametric univariate analysis. Differences between groups were assessed by χ2 test or Fisher-Freeman-Halton’s test, Kruskal-Wallis test, and Wilcoxon-Mann-Whitney-U-test as appropriate.

All tests were two-sided and p-values below 5% were considered significant.

For generating the optimized grouping of the different *IL28B/IFN-L4* haplotypes, the party package of R (R Foundation for Statistical Computing, Vienna, Austria) [28] was used and binary conditional inference classification trees for response were calculated.

For a better association with treatment outcome, non-virological failures (screening failure, treatment discontinuation due to side effects, lost to follow-up) were excluded from statistical analyses summarized in Tables 2, 3, 4, and 5.

**Table 2:** Frequencies of *IFN-L4* and *IFN-L3* genotypes (ss469415590, rs12979860, rs8099917) in HCV genotype 1, 2, 3, and 4 infected patients and different treatment outcomes.

Virologic Response			Total			
Non-Response	Relapse	SVR		*p*		
**GT1(d)**	**C/C**	4 (3.4%)	28 (23.9%)	85 (72.7%)	117 (100%)	
	**C/T**	59 (30.9%)	38 (19.9%)	94 (49.2%)	191 (100%)	
***rs12979860***	**T/T**	27 (45.8%)	8 (13.5%)	24 (40.7%)	59 (100%)	***<0.001^1^***
	**T/T**	29 (14.1%)	45 (21.8%)	132 (64.1%)	206 (100%)	
	**T/G**	50 (35.5%)	28 (19.8%)	63 (44.7%)	141 (100%)	
***rs8099917***	**G/G**	10 (52.6%)	1 (5.3%)	8 (42.1%)	19 (100%)	***<0.001^2^***
	**TT/TT**	4 (3.5%)	29 (25.2%)	82 (71.3%)	115 (100%)	
	**TT/ΔG**	55 (30.4%)	35 (19.3%)	91 (50.3%)	181 (100%)	
***ss469415590***	**ΔG/ΔG**	26 (48.1%)	7 (13.0%)	21 (38.9%)	54 (100%)	***<0.001^1^***
**GT1(t)**	**C/C**	1 (6.7%)	0 (0.0%)	14 (93.3%)	15 (100%)	
	**C/T**	13 (28.3%)	3 (6.5%)	30 (65.2%)	46 (100%)	
***rs12979860***	**T/T**	5 (35.7%)	1 (7.1%)	8 (57.2%)	14 (100%)	***0.197^2^***
	**T/T**	3 (10.3%)	0 (0.0%)	26 (89.7%)	29 (100%)	
	**T/G**	14 (35.9%)	4 (10.3%)	21 (53.8%)	39 (100%)	
***rs8099917***	**G/G**	2 (28.6%)	0 (0.0%)	5 (71.4%)	7 (100%)	***0.018^2^***
	**TT/TT**	1 (6.7%)	0 (0.0%)	14 (93.3%)	15 (100%)	
	**TT/ΔG**	13 (28.3%)	3 (6.5%)	30 (65.2%)	46 (100%)	
***ss469415590***	**ΔG/ΔG**	5 (35.7%)	1 (7.1%)	8 (57.2%)	14 (100%)	***0.197^2^***
GT2	**C/C**	0 (0.0%)	1 (3.7%)	26 (96.3%)	27 (100%)	
	**C/T**	2 (8.3%)	3 (12.5%)	19 (79.2%)	24 (100%)	
*rs12979860*	**T/T**	0 (0.0%)	1 (12.5%)	7 (87.5%)	8 (100%)	***0.269^2^***
	**T/T**	0 (0.0%)	2 (5.4%)	35 (94.6%)	37 (100%)	
	**T/G**	2 (9.5%)	3 (14.3%)	16 (76.2%)	21 (100%)	
*rs8099917*	**G/G**	0 (0.0%)	0 (0.0%)	2 (100%)	2 (100%)	***0.180^2^***
	**TT/TT**	0 (0.0%)	1 (3.7%)	26 (96.3%)	27 (100%)	
	**TT/ΔG**	2 (8.7%)	3 (13.0%)	18 (78.3%)	23 (100%)	
*ss469415590*	**ΔG/ΔG**	0 (0.0%)	1 (12.5%)	7 (87.5%)	8 (100%)	***0.235^2^***
GT3	**C/C**	3 (5.1%)	7 (11.9%)	49 (83.0%)	59 (100%)	
	**C/T**	9 (14.3%)	11 (17.5%)	43 (68.2%)	63 (100%)	
*rs12979860*	**T/T**	5 (27.8%)	1 (5.5%)	12 (66.7%)	18 (100%)	***0.061^2^***
	**T/T**	10 (11.2%)	13 (14.6%)	66 (74.2%)	89 (100%)	
	**T/G**	6 (13.3%)	6 (13.3%)	33 (73.4%)	45 (100%)	
*rs8099917*	**G/G**	1 (16.7%)	0 (0.0%)	5 (83.3%)	6 (100%)	***0.947^2^***
	**TT/TT**	3 (4.9%)	7 (11.5%)	51 (83.6%)	61 (100%)	
	**TT/ΔG**	8 (13.1%)	11 (18.0%)	42 (68.9%)	61 (100%)	
*ss469415590*	**ΔG/ΔG**	5 (27.8%)	1 (5.5%)	12 (66.7%)	18 (100%)	***0.044^2^***
GT4	**C/C**	3 (5.4%)	4 (7.3%)	48 (87.3%)	55 (100%)	
	**C/T**	21 (17.3%)	29 (24.0%)	71 (58.7%)	121 (100%)	
*rs12979860*	**T/T**	13 (36.1%)	5 (13.9%)	18 (50.0%)	36 (100%)	***<0.001^1^***
	**T/T**	15 (10.9%)	22 (16.1%)	100 (73.0%)	137 (100%)	
	**T/G**	20 (27.4%)	20 (27.4%)	33 (45.2%)	73 (100%)	
*rs8099917*	**G/G**	3 (42.9%)	1 (14.2%)	3 (42.9%)	7 (100%)	***<0.001^2^***
	**TT/TT**	3 (5.6%)	4 (7.4%)	47 (87.0%)	54 (100%)	
	**TT/ΔG**	21 (18.3%)	25 (21.7%)	69 (60.0%)	115 (100%)	
*ss469415590*	**ΔG/ΔG**	10 (27.0%)	8 (21.6%)	19 (51.4%)	37 (100%)	***0.002^1^***

GT1(d) – HCV genotype 1 infected patients receiving dual-therapy (PEG-INF + Ribavirin); GT1(t) – HCV genotype 1 infected patients receiving triple-therapy (TPR + PEG-INF + Ribavirin); ^1^p-value from χ2 test; ^2^p-value from Fisher-Freeman-Halton’s exact test.

**Table 3:** Predictors of sustained virologic response (age, HCV RNA concentration, liver fibrosis, *IL28B* rs12979860 CC and rs8099917 TT genotype, and *IFN-L4* ss469415590 TT/TT genotype) in genotype 1, 2, and 3 infected patients.

Predictor	Univariate nonparametric analysis^1^				
	GT1(d)	GT1(t)	GT2/3	GT2	GT3
Lower age	p<0.00	p = 0.41	p = 0.03	p = 0.07	p = 0.02
HCV RNA concentration BL	p = 0.00	p = 0.08	p = 0.11	p = 0.20	p = 0.33
Fibrosis stage	p = 0.00	p = 0.28	p = 0.04	p = 0.09	p = 0.11
rs12979860 CC vs. CT vs. TT	p<0.01	p = 0.29	p = 0.03	p = 0.32	p = 0.06
rs8099917 TT vs. TG vs. GG	p<0.01	p = 0.02	p = 0.69	p = 0.22	p = 0.88
ss469415590 TT/TT vs. TT/ΔG vs. ΔG/ΔG	p<0.01	p = 0.24	p = 0.02	p = 0.30	p<0.05

GT1 (d) – HCV genotype 1 infected patients receiving dual-therapy (PEG-INF + Ribavirin); GT1 (t) – HCV genotype 1 infected patients receiving triple-therapy (TPR + PEG-INF + Ribavirin).

GT – genotype; BL – baseline; n.a. – not applicable.

1 p-values from Wilcoxon-Mann-Whitney-U-test, Kruskal-Wallis-test and χ2 test for quantitative and categorical predictors, respectively.

**Table 4:** Multivariate logistic regression of baseline predictors (age, HCV RNA concentration, liver fibrosis, *IL28B* rs12979860 CC and rs8099917 TT genotype, and *IFN-L4* ss469415590 TT/TT genotype) in genotype 1 infected patients.

Predictor	Multivariate logistic regression GT1(d)					
	beta	SD (beta)	p	Odds-Ratio	95% CI	
Lower age						
HCV RNA concentration BL	0.9339	0.2469	0.0002	2.5445	1.5599	4.1505
Fibrosis stage	0.4744	0.1819	0.0091	1.6071	1.1252	2.2954
rs12979860 CC vs. CT and TT	1.0790	0.3629	0.0029	2.9418	1.4443	5.9917
rs8099917 TT vs. TG and GG	0.6929	0.3228	0.0318	1.9995	1.0622	3.7642
ss469415590 TT/TT vs. TT/ΔG and ΔG/ΔG	-	-	n. s.	-	-	-

GT – genotype; BL – baseline; CI – confidence interval; n. s. – not significant.

**Table 5:** Correlation of biochemical, virologic and histological parameters of genotype 1 (n = 371 dual and n = 81 triple therapy) and 2/3 (n = 198) infected patients with rs12979860, rs8099917, and ss469415590 genotypes.

Variable	rs12979860 CC vs. CT/TT				
	GT1 (d) *P*	GT1 (t) *P*	GT2 *P*	GT3 *P*	GT2/3 *P*
Higher ALT	0.069	0.415	0.026	0.054	0.011
Higher HCV RNA concentration	<0.001	0.007	0.264	<0.001	<0.001
Higher fibrosis stage	0.173	0.336	0.891	0.874	0.850
**Variable**	**rs8099917 TT vs. TG/GG**				
	**GT1 (d) ** ***P***	**GT1 (t) ** ***P***	**GT2 ** ***P***	**GT3 ** ***P***	**GT2/3 ** ***P***
Higher ALT	0.095	0.517	0.099	0.153	0.077
Higher HCV RNA concentration	<0.001	0.339	0.897	0.005	0.017
Higher fibrosis stage	0.032	0.338	0.336	0.399	0.802
**Variable**	**ss469415590 TT/TT vs. TT/ΔG/ΔG/ΔG**				
	**GT1 (d) ** ***P***	**GT1 (t) ** ***P***	**GT2 ** ***P***	**GT3 ** ***P***	**GT2/3 ** ***P***
Higher ALT	0.109	0.336	0.018	0.035	0.006
Higher HCV RNA concentration	<0.001	0.007	0.313	<0.001	<0.001
Higher fibrosis stage	0.796	0.415	0.798	0.947	0.849

Calculation is based on HCV genotype 1, 2, and 3 infected patients with available data on ALT, HCV RNA concentration and fibrosis stage. P-values are from Wilcoxon-Mann-Whitney-U-test and are not corrected for multiple testing.

## Results

### Frequencies of interferon-lambda genotypes

**Figure 1:**
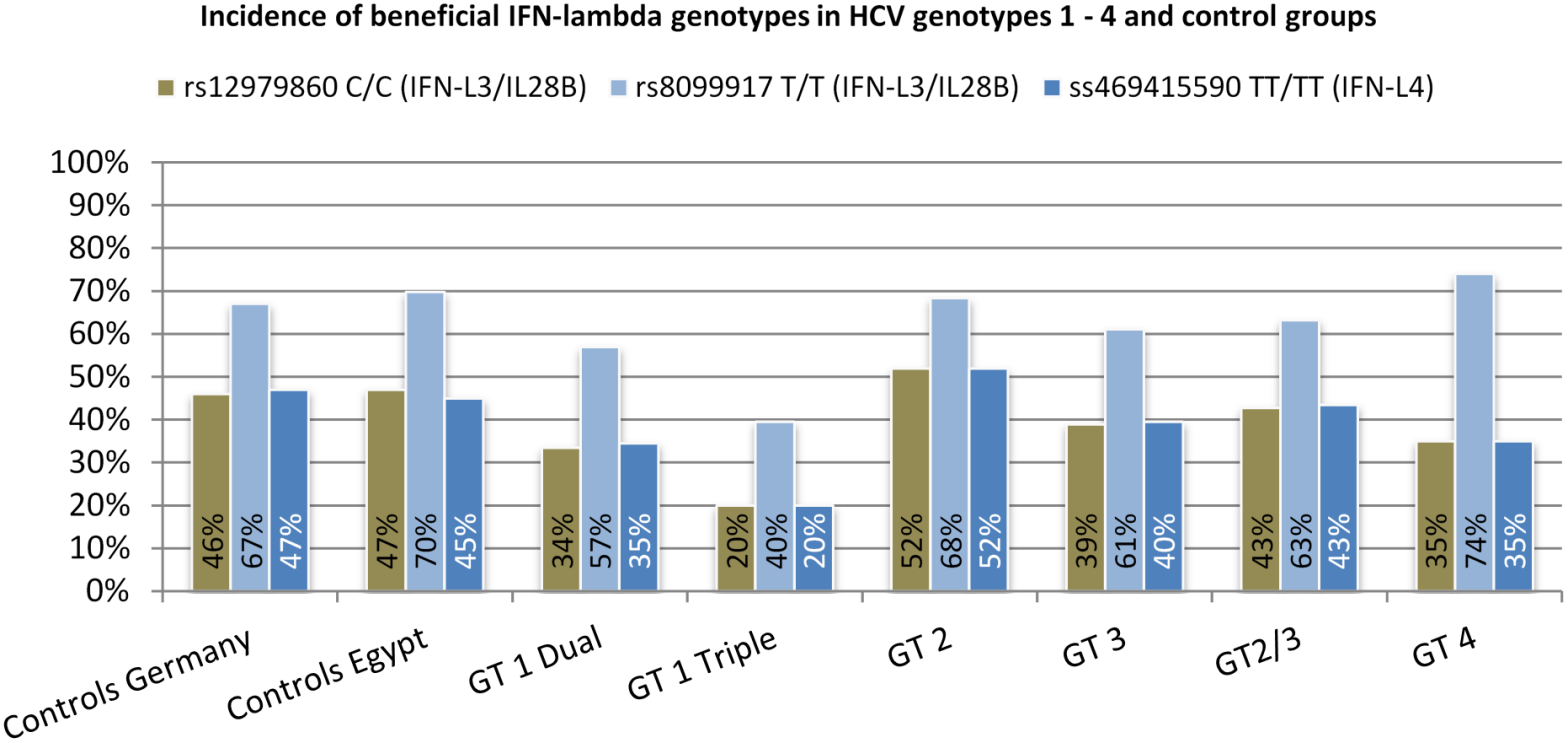
Frequencies of interferon-lambda genotypes in different patient and control cohorts.

Distribution of *IL28B* rs12979860 C/C and *IFN-L4* ss469415590 TT/TT in a German control population was almost similar with 46% and 47%, respectively, whereas *IL28B* rs8099917 T/T existed to a higher extend with 67% (Figure 1). The Egyptian control cohort showed frequencies of the interferon-lambda genotypes comparable with the German control population (47% for rs12979860 C/C, 70% for rs8099917 T/T, and 45% for ss469415590 TT/TT).

For patients with chronic hepatitis C the frequency of the *IL28B* rs12979860 C/C genotype was lowest in the HCV genotype 1 (dual and triple) and in the HCV genotype 4 cohort (34%, 20%, and 35%). The extremely low frequency of 20% in HCV genotype 1 triple therapy patients is explained by a relative large number of previous treatment failure patients in this cohort. Also for the second beneficial *IL28B* genotype (rs8099917 T/T) frequencies were lower in genotype 1 infected patients (57% genotype 1 dual and 40% genotype 1 triple), in comparison with healthy controls, but this is not true for genotype 4 patients, where the incidence of rs8099917 T/T is highest (74%) of all analyzed groups. The frequency of the beneficial *IFN-L4* genotype was almost similar to rs12979860 C/C (35% genotype 1 dual, 20% genotype 1 triple, and 35% genotype 4).

In HCV genotype 2 patients, *IL28B* rs12979860 C/C frequency was slightly increased (52%), whereas in HCV genotype 3 patients a decrease (39%) compared to the German control cohort (46%) could be observed. This is consistent with general higher SVR rates observed in genotype 2 infected patients in comparison to other HCV genotypes. The incidence of *IL28B* rs8099917 T/T was relatively high for both HCV genotypes (68% genotype 2 and 61% genotype 3) and comparable to the control cohorts. In the HCV genotype 2 cohort, the frequency of *IFN-L4* ss469415590 TT/TT was 52% and thus only slightly higher as the control and much higher than in genotype 1, 3, and 4 cohorts. With 40% the HCV genotype 3 cohort showed a slight decrease of *IFN-L4* ss469415590 TT/TT compared to the control group (47%). Frequencies of interferon-lambda genotypes are shown in Figure 1.

### Association of interferon-lambda SNPs with virologic response

**Figure 2:**
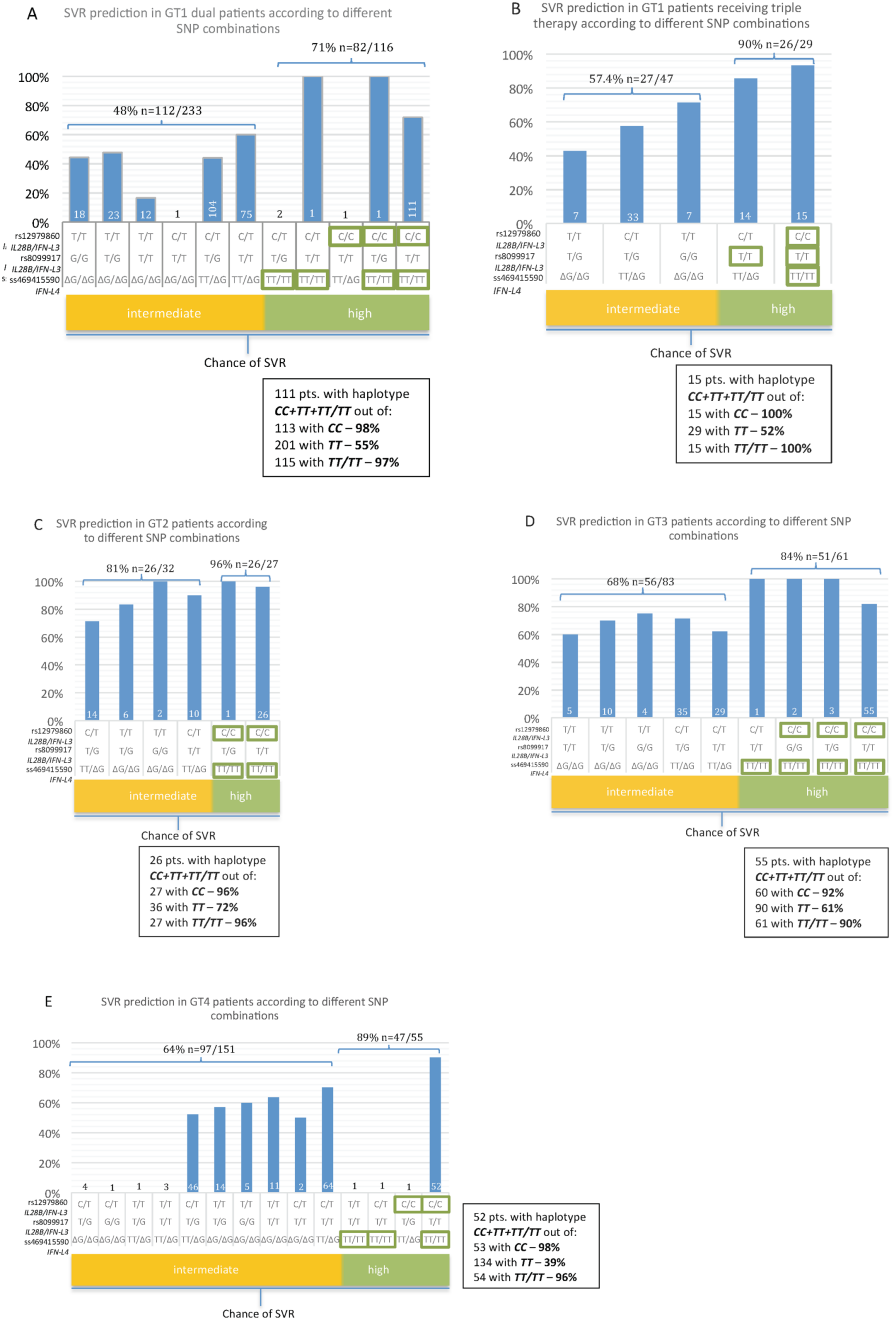
A-2E: SVR prediction rates in HCV infected patients based on interferon-lambda host genotypes and grouping of such by applying a binary regression tree analysis. Figure 2A: HCV genotype 1 patients treated with dual-therapy (PEG-IFN/RBV). Figure 2B: HCV genotype 1 patients treated with triple-therapy (TVR/PEG-IFN/RBV). Figure 2C: HCV genotype 2 patients. Figure 2D: HCV genotype 3 patients. Figure 2E: HCV genotype 4 patients. The boxes show the proportion of the CC+TT+TT/TT haplotype in all patients having rs12979860 CC, rs8099917 TT, and ss469415590 TT/TT, respectively.

As a next step we tried to find out which single SNP and which combination of SNPs in a best way would be able to predict SVR to antiviral therapy, therefore we calculated classification trees. Not all combinations of the different SNPs were observed in the different groups and some combinations were only present in very few patients. Overall, a grouping of SNPs with intermediate and high SVR rates according to the results of the binary regression trees was possible. Analyzing all HCV genotypes and *IFN-L3/IFN-L4* genotypes revealed rs12979860 C/C as the factor, which is highest associated with SVR over all groups. Therefore, all haplotypes including *IFN-L3* rs12979860 C/C were assigned in the “high SVR rate” group. As next step, the groups with different HCV genotypes were analyzed individually. Interestingly, in the vast majority of patients with the *IL28B* rs12979860 C/C genotype also beneficial genotypes of the two other SNPs (*IL28B* rs8099917 T/T and *IFN-L4* ss469415590 TT/TT) were observed (98% in genotype 1 dual, 100% in genotype 1 triple, 96% in genotype 2, 92% in genotype 3, and 98% in genotype 4) (Figures 2A–2E). SVR rates typically were highest in the group with *IL28B* rs12979860 C/C or *IFN-L4* ss469415590 TT/TT (group “high”).

Of note, the new *IFN-L4* ss469415590 genotype was the only significant predictor for SVR in HCV genotype 3 patients (Table 2; Figure 2D). A summary of frequencies of all interferon-lambda genotypes is given in Table 2.

A sub-analysis of HCV genotype 1 and genotype 2 infected patients with F3/F4 fibrosis who received dual therapy showed largely the same results for association of the different IFN-lambda genotypes with SVR. The only differences were seen in the HCV genotype 1 triple therapy and genotype 3 cohorts, where *IL28B* rs8099917 T/T and *IFN-L4* ss469415590 TT/TT, respectively, were no longer significantly associated with SVR (p = 0.779 and p = 0.180). When increasing the sample size by analyzing HCV genotype 2 and 3 together, a trend towards a significant association of *IFN-L4* and treatment outcome (p = 0.077) could be observed.

#### HCV genotype 1 infected patients – dual therapy

In HCV genotype 1 infected patients, treated with dual therapy, all three single beneficial SNPs (C/C, T/T and TT/TT) showed a high correlation with SVR (p<0.001) (Table 2).

For optimization of prediction of SVR, grouping of the different SNPs was performed. The *IL28B* rs12979860 C/C genotype in combination with *IL28B* rs8099917 T/T and *IFN-L4* ss469415590 TT/TT occurred in 98% of all rs12979860 C/C carrying patients. With 71% SVR rate (n = 82/116), the chance of SVR was highest in these patients carrying rs12979860 C/C and/or ss469415590 TT/TT compared to all other interferon-lambda genotype combinations (p = 0.001) (Figure 2A). Among all carriers of rs8099917 T/T, only 55% had the combination with the other two beneficial genotypes (n = 111/201). Without the presence of *IL28B* rs12979860 C/C or *IFN-L4* ss469415590 TT/TT genotype only an intermediate chance to achieve an SVR is given (48%, n = 112/233), (Figure 2A).

Patients in this cohort were treated for different numbers of weeks, according to their on-treatment viral response. To ensure, that these distinctive treatment durations did not have any effect on this analysis, we calculated the association between treatment duration and outcome, where no significant differences could be observed (p = 0.320).

#### HCV genotype 1 infected patients – triple therapy

For triple therapy SVR was correlated with the beneficial *IL28B* rs8099917 genotype as a single polymorphism only (Table 2). Calculation of the classification tree also revealed rs8099917 T/T as strongest predictor for SVR (p = 0.026).

All *IL28B* rs12979860 C/C patients also had the beneficial *IL28B* rs8099917 and *IFN-L4* ss469415590 genotypes. In contrast, only 52% of patients with rs8099917 T/T also show rs12979860 C/C and ss469415590 TT/TT (n = 15/29). The highest SVR rates were observed for patients owing at least one beneficial genotype of the 3 IFN-lambda SNPs, irrespective of which one (90%, n = 26/29). Only an intermediate chance of SVR was given for all other possible combinations of *IL28B* and *IFN-L4* genotypes (57%, n = 27/47). *IFN-L4* genotyping did not confer any advantage in SVR prediction (Figure 2B).

#### HCV genotype 2 infected patients

All single interferon-lambda SNPs showed a limited association with SVR in genotype 2 infected patients (Table 2). All but one patient with rs12979860 C/C were also carriers of the other two beneficial genotypes (rs8099917 T/T and ss469415590 TT/TT) (96%, n = 26/27), whereas 72% of rs8099917 T/T carriers also showed rs12979860 C/C and ss469415590 TT/TT (n = 26/36), this was the highest value in all analyzed patient groups. Patients with an HCV genotype 2 infection and rs12979860 C/C or ss469415590 TT/TT achieved the highest overall SVR rates (96%, n = 26/27). The remaining haplotypes, occurring in 32 patients, led to an accumulated SVR rate of 81% (n = 26/32) (Figure 2C).

#### HCV genotype 3 infected patients

In HCV genotype 3 patients *IFN-L4* ss469415590 TT/TT was the only variant significantly associated with SVR as a single polymorphism (p = 0.044) (Table 2). All three beneficial genotypes occurred in 92% (n = 55/60) of *IL28B* rs12979860 C/C and in 90% (n = 55/61) of *IFN-L4* ss469415590 TT/TT carriers (Figure 2D). This represented the lowest co-incidences of all tested cohorts. In contrast, the portion of *IL28B/IFN-L4* haplotype C/C+T/T+TT/TT carriers out of all rs8099917 T/T harboring patients was relatively high with 61% (n = 55/90) (Figure 2D). Again, highest SVR rates are given for patients with *IL28B* rs12979860 C/C or *IFN-L4* ss469415590 TT/TT alone or in combination (84% n = 51/61). This was consistent with the classification tree result, where *IFN-L4* was calculated as best predictor of SVR, though not reaching statistical significance (p = 0.251). The remaining haplotypes were associated with a 68% chance for achieving sustained response to antiviral therapy (n = 56/83) (Figure 2D).

#### HCV genotype 4 infected patients

For the HCV genotype 4 cohort a good correlation of beneficial single *IL28B* rs12979860 and rs8099917 genotypes with SVR was shown (p<0.001 each), as well as for the beneficial *IFN-L4* SNP (p = 0.002) (Table 2).

Overall, a large number of possible interferon-lambda genotype combinations was observed in genotype 4 patients (n = 14). All but one patient with the beneficial *IL28B* rs12979860 C/C genotype also had the two other beneficial *IL28B/IFN-L4* genotypes (98%, n = 52/53). Comparably, 96% (n = 52/54) of the *IFN-L4* ss469415590 TT/TT carriers had all three beneficial genotypes and the SVR rate was again highest in this group of rs12979860 C/C and/or ss469415590 TT/TT carriers (86%, n = 47/55). Only 39% (n = 52/134) of the patients with rs8099917 T/T were part of the group with all three beneficial genotypes (Figure 2E). From the patients with the remaining haplotypes, 64% (n = 97/151) responded to antiviral therapy (Figure 2E). Classification tree calculation showed *IL28B* rs12979860 C/C as best predictor of SVR, followed by rs8099917 T/T in those patients harboring rs12979860 C/T.

### Association with biochemical, virologic and histological parameters

In 442/466 HCV genotype 1 (367/385 dual-therapy and 75/81 triple-therapy), in 60/79 genotype 2, and in 141/191 genotype 3 patients data on age, baseline viral load, and fibrosis stage were available (Table 3). In the HCV genotype 1 dual-therapy cohort age (p<0.001), HCV RNA concentration at baseline (p = 0.002), and fibrosis stage (p = 0.004) were associated with SVR. None of the analyzed parameters turned out to have any predictive value for the HCV genotype 1 triple therapy cohort and the HCV genotype 2 infected patients when analyzed separately. A lower age (p = 0.021) increases the likelihood for HCV genotype 3 patients to respond to antiviral therapy. When taken together, in HCV genotype 2/3 patients a lower age (p = 0.033) and fibrosis stage (p = 0.036) is associated with virus eradication.

Multivariate analysis of potential baseline predictors of virologic response in HCV genotype 1(d) patients revealed lower baseline HCV RNA concentration, fibrosis stage, and *IL28B* genotypes rs12979860 CC and rs8099917 TT to be significantly associated with SVR (Table 4). Including HCV RNA concentration at week 4, a non-baseline parameter for SVR prediction, in the multivariate analysis, this factor turned out to be strongest associated with SVR (p<0.001), while all baseline predictors dropped out. In HCV genotype 2 and 3 infected patients, none of the parameters remained significantly associated with virologic response in the multivariate analysis.

**Figure 3:**
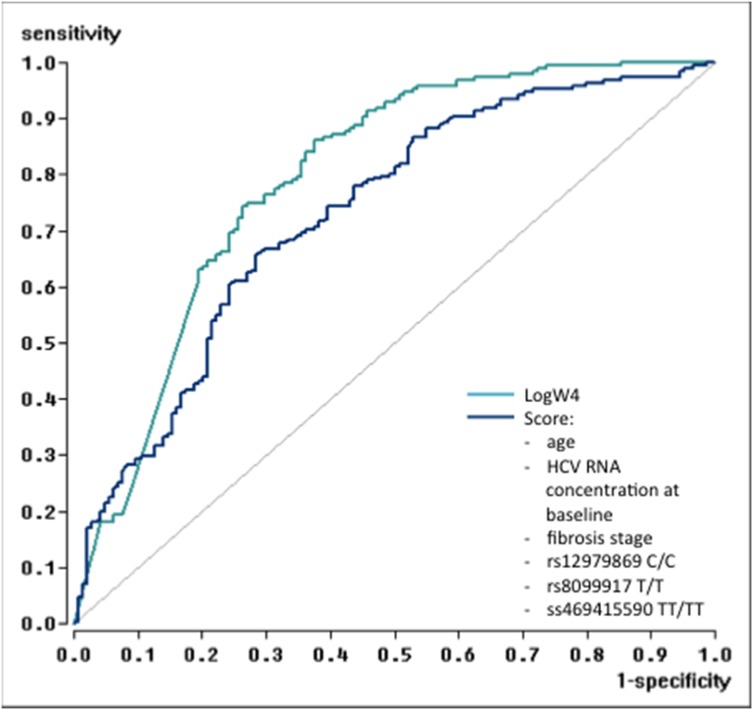
Results of the Delong-test for comparison of ROC-curves (paired) were as follows: Test 1 (VLwk4): AUC = 0.793653, SD = 0.025563, CI = [0.743552; 0.843755]; Test 2 (Score): AUC = 0.729519, SD = 0.028006, CI = [0.674629; 0.784410]. VLwk4 – HCV RNA concentration at week 4 under therapy Score – includes the baseline parameters: age, HCV RNA concentration at baseline, fibrosis stage, rs12979869 C/C, rs8099917 T/T, and ss469415590 TT/TT.

The accuracy of the different models was assessed and compared by the area under the ROC curve (Figure 3).

ALT levels, HCV RNA concentration at baseline and fibrosis stage were correlated with rs12979860, rs8099917, and ss469415590 genotypes. Results are shown in Table 5. An association of *IL28B* rs12979860 C/C with higher ALT values was observed for HCV genotype 2 (p = 0.026) and genotype 2/3 (p = 0.011) patients, when analyzed together. In HCV genotype 2 and 3 patients, the ss469415590 TT/TT genotype was also correlated with higher ALT levels, regardless of being analyzed alone or together (p = 0.018, p = 0.035, and p = 0.006). The rs12979860 C/C and the ss469415590 TT/TT genotype were significantly associated with a higher HCV RNA concentration at baseline in HCV genotype 1(d), 1(t), and 2/3 infected patients as well as in genotype 3 patients alone (p<0.001, p = 0.007, p<0.001, and <0.001 for both SNP genotypes). HCV genotype 2 patients showed no correlation between rs12979860 or ss469415590 genotype and baseline viral load. For fibrosis stage no association was observed at all for both genotypes. *IL28B* rs8099917 T/T was significantly associated with baseline HCV RNA concentration in HCV genotype 1(d) (p<0.001), genotype 3 (p = 0.005), and genotype 2/3 together (p = 0.017). No association could be observed for ALT levels and rs8099917 in any patient group. Only for the HCV genotype 1 dual-therapy cohort an association between fibrosis stage and rs8099917 T/T could be shown (p = 0.032).

## Discussion

The best possible prediction of SVR to antiviral therapy in patients chronically infected with hepatitis C virus is of prime importance. To date, direct antiviral therapies including first and second generation protease inhibitors telaprevir, boceprevir and simeprevir, respectively, and the polymerase inhibitor sofosbuvir represent the first available options for highly effective interferon-containing and interferon-free ways of HCV eradication. In near future, even more direct antiviral agents will be approved for various combinations of interferon-free treatment options. However, in many countries approval and reimbursement of direct antiviral agents will be delayed. Also, dual combination therapies or first generation protease inhibitor-based triple therapies may be cost effective options to achieve an SVR in a subgroup of patients with beneficial predictors of treatment response.

Biochemical, virologic and histological parameters were described to be associated with SVR in HCV genotype 1 infected patients who were treated with PEG-IFN and ribavirin. Definition of these parameters for patients infected with other HCV genotypes and/or receiving direct antiviral drugs is also of great importance [29].

Highest association with response to PEG-IFN and ribavirin therapy was found for a host polymorphism near the *IL28B* gene (rs12979860) in HCV genotype 1 and 2/3 infected patients [13]–[16], [22], [23] so far. Recently, another polymorphism upstream of *IL28B* was found and described as the missing functional variant [24]. This so-called TT/ΔG polymorphism (ss469415590) is in strong linkage disequilibrium with rs12979860 but is featured by an even higher association between spontaneous and treatment-mediated clearance of HCV than reported for *IL28B* rs12979860 [25]. Even in patients treated with sofosbuvir and ribavirin, the ΔG polymorphism was described as associated with slower viral clearance [30]. Type I (IFN-α/β) and III (IFN-λs) interferons are leading to transcription of interferon stimulated genes (ISGs) by binding to their receptors and subsequent activation of the Jak-STAT pathway [31], [32]. Recently, it was shown, that the TT/ΔG polymorphism but not rs12979860 or rs8099917 in PBMCs was associated with induction of ISGs like IP-10 on simulation of viral infection by poly(I:C) [24]. Thereby, a functional association between TT/ΔG polymorphism and host immune response was assumed [24]. In line with these data in patients with a poor response to PEG-IFN/ribavirin therapy, high pre-treatment concentrations of ISGs including IP10 were observed. In addition, ISGs were significantly higher expressed in HCV genotype 1/4 patients compared to HCV genotype 2/3 patients [33]. While there is a clear correlation between allelic variants of IL28B and IFN-L4 and the induction of ISGs in the liver, the mechanism which leads to ISG up-regulation in patients with poor response to interferon-based antiviral therapy is not fully understood [24], [34]–[36]. Most recently, an association of enhanced IFN-alfa induced IFN lambda receptor 1 (IFN-lR1) expression with minor IL28B genotypes was observed pointing to a potential indirect mechanism [37].

In the present study, the potential as predictor for treatment outcome of the TT/ΔG polymorphism in patients with different HCV genotypes (1, 2, 3, and 4) receiving dual (PEG-IFN + ribavirin) or triple (TPR + PEG-IFN + ribavirin) therapy was investigated.

We could find an almost equal distribution of the TT/TT variant when compared to the favorable *IL28B* SNP rs12979860 CC. The proportion of treatment naïve patients homozygous for CC and TT/TT increased from HCV genotype 1/4 (34–35% and 35%) over genotype 3 (39% and 40%) to genotype 2 infected patients (52% and 52%) and healthy controls (46–47% and 45–47%) which seems to be correlated to the SVR rates increasing also from HCV genotype 1/4 over 3 to 2 [16]. While in the majority of patients a coincidence of beneficial CC and TT/TT SNPs was detected (>90%) importance of single presence of these beneficial SNPs together with other *IL28B/IFN-L4* polymorphisms is unclear.

For analysis of a correlation of the ss469415590 polymorphism as single SNP with SVR, in the present study a significant association was seen for HCV genotype 1, genotype 3, and genotype 4 patients who received dual therapy but not for genotype 2 patients as well as HCV genotype 1 patients who received triple therapy. In the study by Bibert et al., data was analyzed for genotype 1+4 and for genotype 2+3 together [24]. Here, ss469415590 TT/TT was associated with SVR in both patient groups, while rs12979860 CC did not allow significant prediction of SVR in genotype 2/3 patients. Unfortunately, no statement to the distribution of genotype 2 and 3, respectively, within the total number of 252 patients was given. Similar to our study, Stättermayer et al. [38] postulated an association between *IFN-L4* and SVR for the groups of HCV genotype 1 as well as 4 infected patients. However, also in this study genotype 2 (n = 23) and 3 (n = 185) infected patients were analyzed as one group and no association of *IFN-L4* genotype with SVR was observed. Results of the present study support evidence of significant differences of interferon responsiveness between genotype 2 and 3 infected patients. While for HCV genotype 2 patients none of the single SNPs including IFN-L4 showed a significant association with treatment outcome, patients with HCV genotype 3 could benefit from ss469415590 genotyping, as *IFN-L4* ss469415590 TT/TT was significantly associated with SVR.

In addition to correlation of single *IL28B* and *IFN-L4* SNPs as in previous studies [24], [38], in the present study we aimed to improve SVR prediction by combination of different SNPs in order to identify distinct haplotypes associated with virologic treatment response.

For clinical practice a stepwise analysis of IFN-lambda SNPs in single patients could be recommended for an optimized virologic and economical management.

For treatment with dual combination of PEG-IFN and ribavirin a high likeliness of SVR was observed only in patients with either beneficial rs12979860 C/C or ss469415590 TT/TT genotypes or a combination of both. In genotype 1, 2, 3, and 4 infected patients SVR rates are 71%, 96%, 84%, and 89%, respectively. Due to the higher antiviral efficacy it was not surprising that all combinations with beneficial *IL28B* (rs12979860 C/C and rs8099917 T/T) and *IFN-L4* (ss469415590 TT/TT) SNPs were associated with a high chance of SVR on TVR/BOC based triple therapy. Here, SVR rates were 100% for treatment naïve and relapsers and 63% for previous non-responders. Overall, by this strategy of selection of patients with beneficial IFN lambda SNP genotypes, 33% of treatment naïve patients could have been selected for dual or triple therapy with first generation PIs with excellent SVR chances.

A potential scenario would be restriction of antiviral therapy to patients with advanced disease. Overall the subgroup of patients with F3 fibrosis or cirrhosis was limited in the present study (14–52%). However, in the largest group of genotype 1 infected patients who received PEG-IFN, ribavirin combination therapy also in the subgroup with advanced fibrosis/cirrhosis the overall association of SVR with IFN-lambda polymorphisms remained identical.

In addition to prediction of SVR also treatment duration may be important for selection of IFN-based versus –free treatment options. For telaprevir triple therapy, 68% of treatment naïve and relapse patients with excellent SVR chances based on *IL28B* and *IFN-L4* haplotype also received only 24 weeks treatment, while for IFN and ribavirin combination therapy shortening of treatment duration to 24 weeks was possible in 21% of patients with beneficial IFN-lambda SNPs only.

Taken together, differential and stepwise analysis of IFN-lambda gene SNPs in HCV infected patients with different genotypes may help to identify subgroups of patients for an optimized selection of antiviral therapy. Given a positive prediction of SVR between 71% and 96% based on proposed *IL28B*/*IFN-L4* haplotypes with high chances of HCV eradication selection of IFN-based antiviral therapies may be an option for settings with economical limited resources, although long treatment durations and side effects of first generation protease inhibitors represent limitations. Also inclusion of a confirmation cohort was not possible in the present study and thus results have to be confirmed by independent data. However, our approach may also be applied for newer IFN-based triple therapies with simeprevir and other DAAs. Administration of more expensive DAA regimens could so be restricted to subgroups of more difficult to treat patients with overall SVR rates around 90% for the entire group.
